# Organic Molecules are Involved in Ni-Struvite Biomineralization by *Streptomyces mirabilis*

**DOI:** 10.1007/s12033-026-01580-3

**Published:** 2026-05-15

**Authors:** Flávio Silva Costa, Thomas Krüger, Nico Ueberschaar, Olaf Kniemeyer, Axel Brakhage, Erika Kothe

**Affiliations:** 1 Institute of Microbiology, Friedrich Schiller University Jena, 07743 Jena, Germany; 2 Molecular and Applied Microbiology, Leibniz Institute for Natural Product Research and Infection Biology-Hans Knöll Institute, Jena, Germany; 3 Mass Spectrometry Platform, Faculty for Chemistry and Earth Sciences, Friedrich Schiller University, Jena, Germany

**Keywords:** Biomineralization, Biomimetic mineral formation, *Streptomyces*, *Struvite*, Nickel, Proteome, Metabolome

## Abstract

The Gram-positive, filamentous soil bacterium *Streptomyces mirabilis* P16B-1 has been shown to tolerate high environmental concentrations of Ni. The extremely high metal tolerance is partly provided through biomineralization, observed for both Mg bearing struvite and Ni-struvite where Ni ions replace Mg in the mineral lattice. The minerals showed different morphologies. This led us to assume that excreted substances may have the capacity to influence the mineral formation process that occurs at a distance to the cells. Here, we could show with metabolomics and proteomics studies that 23 metabolites as well as 18 proteins potentially co-precipitate with the minerals. Secreted secondary metabolites reduced in the supernatant after mineralization were identified as ergothioneine, diacetyllegionaminic acid, nevaltophin F, and diaminonaphthalene. The compounds phenylacetaldehyde and margaric acid also reduced after mineral precipitation did not alter mineral macromorphology. In addition, ten proteins that co-precipitated with statistical significance were predicted to be secreted. Among those, a specific nickel binding protein, NikA, was reduced in high amounts from the supernatant upon crystal formation. The potential of secreted organic molecules and proteins to bind specific surfaces of the mineral thereby redirecting crystal growth in a microbially influenced biomineralization process. Implications for biotechnological metal-struvite use are discussed.

## Introduction

Biomineralization is known to provide structural functions [1], nutrient storage [2, 3], protection against toxic compounds [4, 5], or predators [6] for the producing organism. Some of these functions extend to other organisms in a community beyond the one responsible for mineral formation. For example, immobilizing nutrients that otherwise would be washed out could provide such a beneficial trait to the community [7]. Additionally, due to a high biomineralization rate, biomineral formation has a major impact on biogeochemical cycles, including metals [8]. Furthermore, it has been explored biotechnologically [9]. In addition to struvite being used to re-gain phosphorus and nitrogen from wastewaters, cobalt- or nickel-containing struvite has been implicated in circular economy for precious metal retrieval [10, 11].

Despite biomineralogy having been known for a long time, the mechanisms behind molecular traits involved in mineral formation are not fully understood. Biomineralization is a complex process difficult to observe in situ and the information obtained from mineral analyses is limited [12]. However, understanding the mechanism of mineralization is important when aiming to apply biomineralization for biotechnological use, or understanding what drives community composition in a specific habitat [13]. The two main concepts involve microbially controlled processes with full control over the precipitation, e.g., intracellular magnetite crystallization, and the microbially induced mineralization thought to be related to microbes controlling the abiotic conditions, e.g., by changing the pH/Eh near the cell [14]. However, for an inorganic mechanism of induced precipitation, the morphology of crystals would still be unchanged from abiotic conditions, which often is not the case [4, 15]. This led to more elaborate concepts like the description of non-classical nucleation. Here, the process proceeds from a (locally) supersaturated solution over formation of several nucleation points that lead to micro-clusters which then can aggregate. From a metastable phase, phase transition leads to the final crystallization that may be controlled by the presence of microbial metabolites or proteins (compare [16, 17]). While for nucleation, a non-specific organic–inorganic interaction is envisioned, while, for the later steps, regulating the final shape. Often extracellular polymers like the extracellular matrix, cell wall components of the outer membrane of Gram-negatives have been named. Biomineralization may occur due to the interaction of ions with organic molecules, e.g., bacterial cell surfaces providing carboxylates, amines, and phosphates that interact with metal ions and facilitate the nucleation of minerals [18, 19] and the presence of a surface may decrease the energy necessary to stabilize the nucleus [20]. First reports suggested that intrinsically disordered proteins may be involved in stabilizing the micro-clusters which would allow for the easier transition into stable phases [17, 20]. Here, clearly a more molecular understanding is needed.

Excreted organic molecules such as peptides [21–23], siderophores [24], soluble extracellular polymeric substances [25], or other organic materials [26] have been discussed to provide nucleation triggers. Proteins have a well described role in biomineralization, especially in higher organisms that use protein matrices to control extracellular mineral precipitation [27, 28]. In bacteria, proteins can also be involved either directly in mineral formation [29, 30], or indirectly as stabilizing agents [31–33].

*Streptomyces mirabilis* P16B-1 was isolated from a former uranium mine, and it tolerates extremely high concentrations of nickel and other heavy metals [34]. Additionally, the formation of struvite and Ni-struvite by this filamentous actinobacterium has previously been described, including the formation of higher amounts of crystals with increasing nickel concentrations and different macro-morphologies depending on culture supernatant of the metal-resistant bacterium [35, 36]. Biomineral formation in this strain was associated with nickel tolerance [37] and has been described as a biologically induced mineralization.

Here, we hypothesized that different macro-morphologies of minerals (see [4, 15]) are biologically induced. This is postulated to provide an intermediate biomineralization process for which additional factors controlled by the bacteria are provided. One potential mechanism is the excretion of organic molecules or proteins, which preferentially bind to one face of the developing crystal, thereby redirecting crystal growth. In this scenario, large quantities of the biomolecule(s) are required, making candidates accessible by studying metabolites or proteins before and after precipitation was induced [38]. Understanding the mechanism by which *S. mirabilis* P16B-1 controls biomineral formation can be useful, not only for elucidating a complex and important ecological process but also for opening the door for biotechnological applications including struvite mineralization in wastewater treatment [39]. Additionally, the formation of metal-containing minerals can be used for bioremediation of metal contaminated sites [40]. Therefore, the aim of this work was to investigate the role of organic molecules as well as proteins in the biomineralization of struvite and Ni-struvite by *S. mirabilis* P16B-1.

With that, we can provide evidence for a three-step model that involves initiation of mineralization with a predefined macromorphology. The minerals are forming at a distance from the cells by microbially induced mineralization. Adding to that concept, however, a specific influence of the cells allows for an active influence on the process of biomineral formation.

## Materials and Methods

### Materials Used

The growth medium tryptic soy broth (TSB) was obtained from Becton Dickinson (Heidelberg, Germany), membrane spin filters for 10 kDa cut-off (polyethersolfon, PES; molecular weight cut-off, MWCO) were obtained from VWR (Darmstadt, Germany) and 0.02 µm PES membrane sterile filters as well as high-performance liquid chromatography (HPLC) grade chemicals acetonitrile, margaric acid (heptadecanoic acid, CAS 506—12—7), N,N-dimethylformamide, trichloroacetic acid, triethylammonium bicarbonate, 2,2,2.trifluoro ethanol, ethylacetate, tris(2-carboxyethyl)phosphine, 2-chloroacetamide, and acetone were obtained from Sígma-Aldrich/Merck (Darmstadt, Germany). Chromatography equipment C-18 RP chromatography column, Acclaim Pep Map 100 column, and analytical Acclaim Pep Map RSLC nanocolumn were obtained from (Thermo Fisher Scientific, Waltham, USA), while Amicon Ultra-15 centrifugal filter unit was obtained from Millipore (Burlington, USA).

### Sample Preparation

*Streptomyces mirabilis* P16B-1 was cultivated in TSB and TSB amended with 10 mM NiSO_4_. Supernatant samples were collected by filtration, using 0.02 µm PES membrane sterile filters, after one week of growth. A fraction was collected and further treated for metabolome or proteome analysis before mineral precipitation. The rest of the sample was frozen at −20 °C and afterward thawed at room temperature (21 °C) to induce struvite precipitation. Minerals were removed from the sample by sterile filtering again and the supernatant was further treated as samples after mineral precipitation. All samples were prepared with three biological and three technical replicates (*n* = 3), the use of the same sample before and after precipitation allowed for a direct comparison of the remaining amounts of a compound or a protein in the supernatant after precipitation.

### Metabolomics

Metabolites were extracted with 2:1 methanol:sample (v/v). Ultra-high-performance liquid chromatography coupled with high-resolution mass spectrometry (Ultimate HPG-3400 RS binary pump and WPS-300 auto sampler, Thermo, Bremen, Germany) was used for metabolomics identification of compounds with the temperature set to 10 °C. For sample injection and sampling, a 25-µL injection syringe and a 100 µL sample loop were used. A Thermo Accucore C-18 RP chromatography column (100 × 2.1 mm; 2.6 µm) was used, housed in the TCC-3200 column compartment, at 25 °C. After equilibration for 0.2 min (eluent A, water with 2% acetonitrile and 0.1% formic acid), a gradient for elution (0—100% eluent B, acetonitrile) was followed with 100% eluent B from 8 to 11 min at constant 0.4 mL/min flow rate. The system was re-equilibrated to the initial conditions from 11.1 to 12 min.

### LC–MS/MS

To obtain mass spectra, a Thermo QExactive Plus Orbitrap mass spectrometer (Thermo Fischer, Darmstadt, Germany) was utilized, coupled with a heated electrospray source. The column flow was adjusted at 0.5 min, diverting it from waste to the mass spectrometer, and then at 11.5 min, redirecting it back to waste to prevent contamination of the source. Two full scan modes were employed for monitoring, employing the following parameters. In positive polarity, the scan range was set from 80 to 1200 m*/z*, with a resolution of 70,000. The automatic gain control (AGC) target was set to 3 × 106, and the maximum injection time was 200 ms. General settings included a sheath gas flow rate of 60, an auxiliary gas flow rate of 20, and a sweep gas flow rate of 5. The spray voltage was maintained at 3.0 kV, while the capillary temperature was set to 360 °C. The S-lens radiofrequency (RF) level was adjusted to 50, and the auxiliary gas heater temperature was maintained at 400 °C. The acquisition time frame for data collection spanned from 0.5 to 11.5 min. For the negative mode, all values remained the same except for the spray voltage, which was adjusted to 3.3 kV.

To ensure the reliability of the system, a pooled sample, which consisted of an equal mixture (*v/v*) of all individual samples, was measured at the beginning and end of the experiment. Additionally, this pooled sample was utilized to acquire additional mass spectrometry (MS2) spectra in data-dependent mode, employing the following settings: auto scan range, resolution of 17,500, automatic gain control (AGC) target of 3 × 106, maximum injection time of 32 ms, loop count of 5, preferred charge state of 1, and dynamic exclusion of 30 s.

### Metabolome Analysis

*S. mirabilis* P16B-1 was cultivated in TSB or TSB amended with 10 mM NiSO_4_. Samples were produced in four replicates. Supernatant samples were collected by filtration, using 0.02 µm PES membrane, after one week of growth. A fraction was collected and further treated for the analysis of the metabolome before mineral precipitation. After precipitation, the second fraction was obtained for comparison of data before and after mineral precipitation.

For data analysis, Thermo Compound Discoverer Software 3.2.0.421 was employed, utilizing a metabolomics workflow. The software searched various databases including ChemSpider, *mz*Cloud, *mz*Vault, KEGG, and Metabolika Pathways Databases for sum formula annotation. Furthermore, an in-house database was utilized, which contained additional retention time information based on the Sigma Aldrich (Taufkirchen, Germany) mass spectrometry metabolite library (MSMLS). This in-house database comprised over 600 primary metabolites from various classes such as carboxylic acids, amino acids, amines, polyamines, nucleotides, coenzymes, vitamins, saccharides, fatty acids, lipids, steroids, and hormones. The purpose of this database was to aid in the annotation of potential marker compounds. The software integrated the ion currents and compared the signals from each measurement. This allowed for the calculation of *p*-values for statistical analysis and the determination of ratios between experiments.

### Mineral Synthesis

To test the influence of phenylacetaldehyde and margaric acid in the mineralization of struvite, the mineral was synthesized using an ammonia diffusion system as described by Li [41]. Margaric acid solution was prepared by dissolving margaric acid in dimethylformamide to a concentration of 25 mg/mL. For the analyses, 24-well plates were used. The TSB media containing 0.5% (v/v) phenylacetaldehyde and margaric acid solution were used for filling the wells, and the plate was covered with parafilm. Each well was then aerated by a needle hole that was made in the parafilm covering each well. As control, TSB medium without additional compounds, as well as TSB containing 0.5% of dimethylformamide, was used. The plate was placed in a desiccator containing a beaker with ammonia solution (30%). The samples were incubated at room temperature for one week and minerals were collected afterward.

### Protein Analyses

Proteins of the sterile filtered, cell-free culture supernatant before or after precipitation of minerals from three biological replicates were precipitated in three technical replicates each using 25% trichloroacetic acid (*v/v*). The protein pellet was washed with acetone (100%) and dried. Precipitates were resolubilized in 50 mM TEAB (triethyl ammonium bicarbonate) in 1:1 trifluoroethanol:H_2_O (v/v). Subsequently, cysteine thiols were reduced and carbamidomethylated in one step for 30 min at 70 °C by the addition of each 2 µL of 500 mM tris(2-carboxyethyl)phosphine and 625 mM 2-chloroacetamide. The samples were further cleaned by MeOH/chloroform/H_2_O precipitation following the protocol of Wessel and Flügge [42].

### Protein Digestion

Protein precipitates were resolubilized in 5% trifluoroethanol of 100 mM TEAB in *A. dest.* and digested overnight (18 h) with a Trypsin + LysC mixture at a protein to protease ratio of 25:1. Peptides were cleaned by washing with water saturated ethylacetate [43]. The samples were then evaporated to dryness in a vacuum concentrator (Eppendorf, Leipzig, Germany) and resolubilized in 30 µL of 0.05% trifluoroacetic acid in H_2_O/acetonitrile 98/2 (v/v) and filtered through PES 10 kDa MWCO membrane spin filters. The filtrate was transferred to HPLC vials and injected into the LC–MS/MS (liquid chromatography tandem mass spectrometry) instrument.

### Proteome Analysis

Each sample was measured in triplicate (three analytical replicates of three biological replicates). LC–MS/MS analysis was performed on an Ultimate 3000 nanorapid separation liquid chromatography (RSLC) system connected to a hybrid quadrupole orbitrap (QExactive HF mass spectrometer, Thermo Fisher Scientific, Waltham, USA). Peptide trapping for 5 min on an Acclaim Pep Map 100 column (2 cm × 75 µm, 3 µm) at 5 µL/min was followed by separation on an analytical Acclaim Pep Map RSLC nanocolumn (50 cm × 75 µm, 2 µm). Mobile phase gradient elution of eluent A (0.1% (v/v) formic acid in water) mixed with eluent B (0.1% (*v/v*) formic acid in 90/10 acetonitrile/water) was performed using the following gradient: 0 min at 4% B, 5 min at 8% B, 30 min at 12% B, 75 min at 30% B, 85 min at 50% B, 90—95 min at 96% B, and 95.1—120 min at 4% B. Positively charged ions were generated at spray voltage of 2.2 kV using a stainless steel emitter attached to the Nanospray Flex Ion Source (Thermo Fisher Scientific, Waltham, USA). The quadrupole/orbitrap instrument was operated in full MS/data-dependent MS2 Top15 mode. Precursor ions were monitored at *m/z* 300—1500 at a resolution of 120,000 FWHM (full width at half-maximum) using a maximum injection time (ITmax) of 120 ms and an AGC (automatic gain control) target of 3 × 106. Precursor ions with a charge state of z = 2—5 were filtered at an isolation width of *m/z* 1.6 amu for further higher-energy collisional dissociation (HCD) fragmentation at 28% normalized collision energy (NCE). MS2 ions were scanned at 15,000 FWHM (ITmax = 100 ms, AGC = 2 × 105). Dynamic exclusion of precursor ions was set to 25. The LC–MS/MS instrument was controlled by Chromeleon 7.2, QExactive HF Tune 2.8 and Xcalibur 4.0 software. The mass spectrometry proteomics data are available at the ProteomeXchange Consortium via the PRIDE partner repository (https://www.ebi.ac.uk/pride; dataset identifier PXD049698).

#### Protein Database Search

Tandem mass spectra were searched against the *Streptomyces mirabilis* P16B-1 genome (GenBank accession numbers CP074102 and CP074103) using Proteome Discoverer (PD) 2.4 (Thermo Fisher Scientific, Waltham, USA) and the algorithms of Mascot 2.8 (Matrix Science, Horsham, USA), Sequest HT (version of PD2.4), MS Amanda 2.0, and MS Fragger 3.5. Two missed cleavages were allowed for the tryptic digestion. The precursor mass tolerance was set to 10 ppm and the fragment mass tolerance was set to 0.02 Da. Modifications were defined as dynamic Met oxidation, phosphorylation of Ser, Thr, and Tyr, protein N-term acetylation and/or loss of methionine, as well as static Cys carbamidomethylation. A strict false discovery rate (FDR) of < 1% (peptide and protein level) and a search engine score of > 30 (Mascot), > 4 (Sequest HT), > 300 (MS Amanda), or > 8 (MS Fragger) were required for positive protein hits. The Percolator node of PD2.5 and a reverse decoy database was used for *q*-value validation of spectral matches. Only rank 1 proteins and peptides of the top scored proteins were counted. Label-free protein quantification was based on the Minora algorithm of PD2.4 using the precursor abundance based on intensity and a signal-to-noise ratio > 5. Imputation of missing quantitative values was applied by using abundance values of 75% of the lowest abundance identified per sample. Differential protein abundance was defined as a fold change of > 2, ratio-adjusted *p*-value of < 0.05 (*p-*value/log4ratio), and at least identified in 2 of 3 replicates of the sample group with the highest abundance (prevalence filter).

#### Determination of Element Concentrations

As an indirect measurement of mineralization, the concentrations of Mg, P, and Ni were determined on the supernatant of cultures before and after mineralization. The supernatant of 1-week-old cultures was collected, and mineral precipitation was induced as described above. To investigate the role of proteins in the mineralization process, the samples were also subjected to ultrafiltration using an Amicon Ultra-15 centrifugal filter unit with a nominal molecular weight cut-off (NMWCO) of 30 kDa (Sigma Aldrich, Taufkirchen, Germany). The filtered fraction also underwent a freeze–thaw cycle to induce mineral precipitation and elements concentrations were determined before and after mineralization. Measurements were made using simultaneous radial inductively coupled plasma optical emission/mass spectrometry (ICP-OES/MS) spectrometers (725ES with CCD detector, Agilent, Waldbronn, Germany).

#### Statistical Analysis

All statistical analyses and visualization were conducted in R. Plots were obtained with the packages ggplot2, ggfortify, gggenes, and ggpubr. The normality of the data was assessed using both a *q—q* plot and the Shapiro–Wilk test. Subsequently, appropriate statistical tests were employed based on the distribution of the data. In cases where the data followed a normal distribution, ANOVA and *T*-tests were conducted. When the data did not meet the assumption of normality, equivalent non-parametric tests were utilized. The Kruskal–Wallis test by ranks was employed for multiple comparisons, and the Wilcoxon rank sum test was utilized for pairwise comparisons.

The data for metabolomics and proteomics were treated separately. For metabolites, the database compound identification allowed the annotation of potential marker compounds. With integrated ion currents, the signals from each measurement could be compared. This allowed *p-*value and ratios determination between experiments.

Proteins identified were assessed using R/RStudio workflow (total protein normalization per replicates, Welch test for *p-*value calculations) looking for precipitated protein abundance and the relative percentage of precipitation per protein. High co-precipitation (at least 50%) was screened and proteins with high abundance in the cell supernatant were further evaluated. For selected proteins, encoding genes were identified and scanned using SignalP (https://services.healthtech.dtu.dk/services/SignalP 6.0/; last access 16.4.2024) for excretion prediction.

To select relevant changes, the filter applied for the proteomics analyses were -fold change before/after precipitation with Ni < −2, without Ni > −2, adjusted *p-*value < 0.01, mean abundance before precipitation with nickel of > 1 * 109. Data are available via ProteomeXchange with identifier PXD049698.

#### Genomic Analysis

The identification of the *nur* repressor in *S. mirabilis* P16B-1 was made using the tblastn tool in NCBI [44] using the genome of this strain available in GenBank (Accession No. CP074102.1). For the prediction of signal peptide sequences, SignalP 6.0 [45] was used. Multiple sequence alignments of sequences available in GenBank were obtained with the tool MUSCLE available in Unipro UGENE [46].

## Results

### Formation of Struvite and Ni-Struvite in Abiotic and Biologically Influenced Conditions

**Fig. 1:**
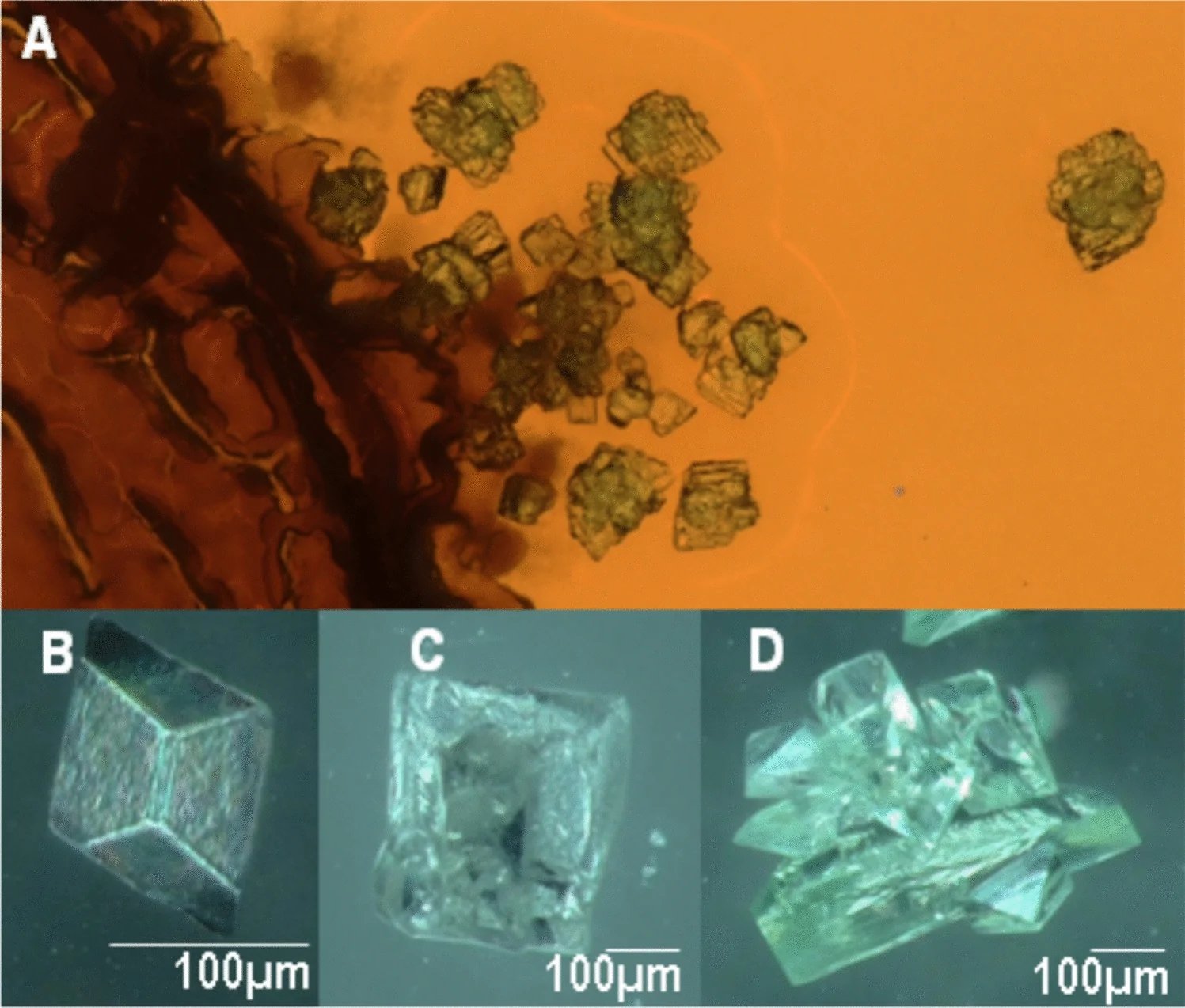
Ni-struvite crystals formed by *S. mirabilis* P16B-1. **A** Green Ni-struvite crystals formed on solid media containing 10 mM NiSO_4_ directly on the bacterial mycelia (brown, left side of panel) as well as apart from the biomass. **B**,**D** Variability of macro-morphologies of crystals formed in liquid media without (**B**), with 5 mM (**C**), and with 10 mM NiSO_4_ (**D**)

The formation of both the magnesium containing struvite and Ni-struvite replacing Ni for Mg in the ammonium phosphate mineral is possible by the same *S. mirabilis* P16B-1 [37]. The minerals were not only formed directly connected to the bacterial mycelium but occurred also at a distance from the cells visible with solid media, suggesting influence of secreted substances (Fig. 1A). The minerals varied from rectangular to rosette-like and composite morphologies formed in liquid media (Fig. 1B-D). The difference between the Ni and Mg containing biominerals is only attributed to the presence of sufficient (≥ 5 mM) Ni in the medium. While the Mg-struvite minerals were needle-like, the Ni containing mineral phases showed a large variety of different shapes (Online Resource 1). Using sterile filtered culture supernatant for induction of new mineral precipitation allowed us to identify molecules coprecipitating with the minerals.

**Fig. 2:**
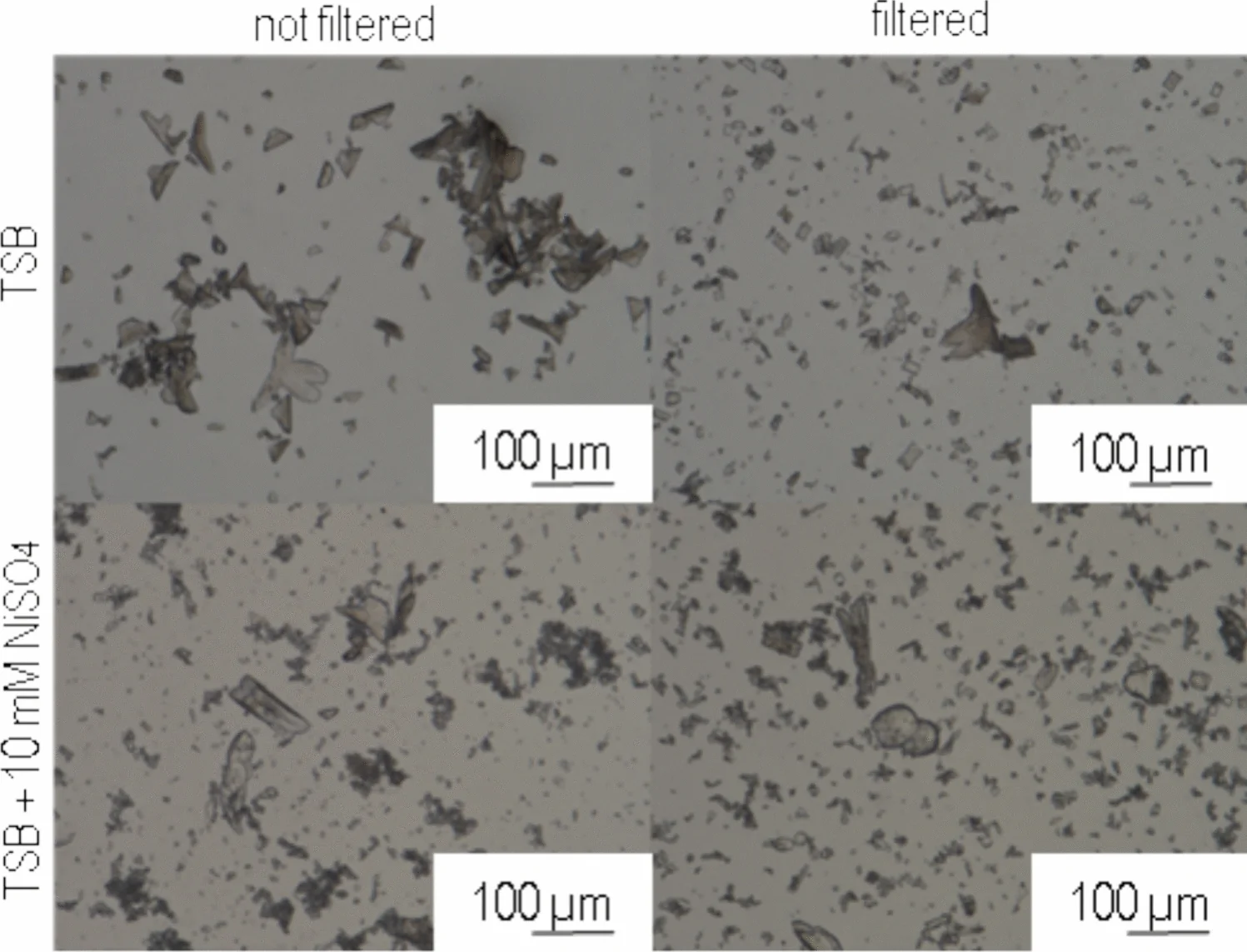
Crystals formed in the supernatant of *S. mirabilis* P16B-1. Conditions with and without addition of nickel salt to the growth medium were compared. In addition, pretreatment by size exclusion filtering removing compounds larger 3 kDa was compared to non-filtered supernatant. Each supernatant sample was then subjected to freeze–thaw-induced mineralization and shown by microphotography

In a first approach, we compared the native supernatant and one which had been filtered to remove molecules larger than 3 kDa. The precipitation of minerals was possible in either case, showing that the mineralization process itself was not dependent on large, secreted molecules (Fig. 2). This opened the road to compare growth with or without Ni for metabolic processes to evaluate, which excreted organic molecules potentially participate in the process.

### Co-precipitation of Metabolites with Biominerals

To visibly impact Ni-struvite morphology, a large amount of any given molecule in the supernatant needs to co-precipitate with the mineral. This hypothesis led us to analyzed metabolites and their quantities before and after precipitation from an unbiased study. A distinct separation was observed between the metabolome of *S. mirabilis* P16B-1 under nickel treatment and without the metal using principal component analysis (Online Resource 2). At the same time, no obvious separation between the same samples obtained before and after struvite precipitation was found with the overall analysis of the metabolomes. This allowed us to directly compare the metabolites in each sample before and after precipitation thereby directly linking the compound’s relative presence to biomineral formation.

**Fig. 3:**
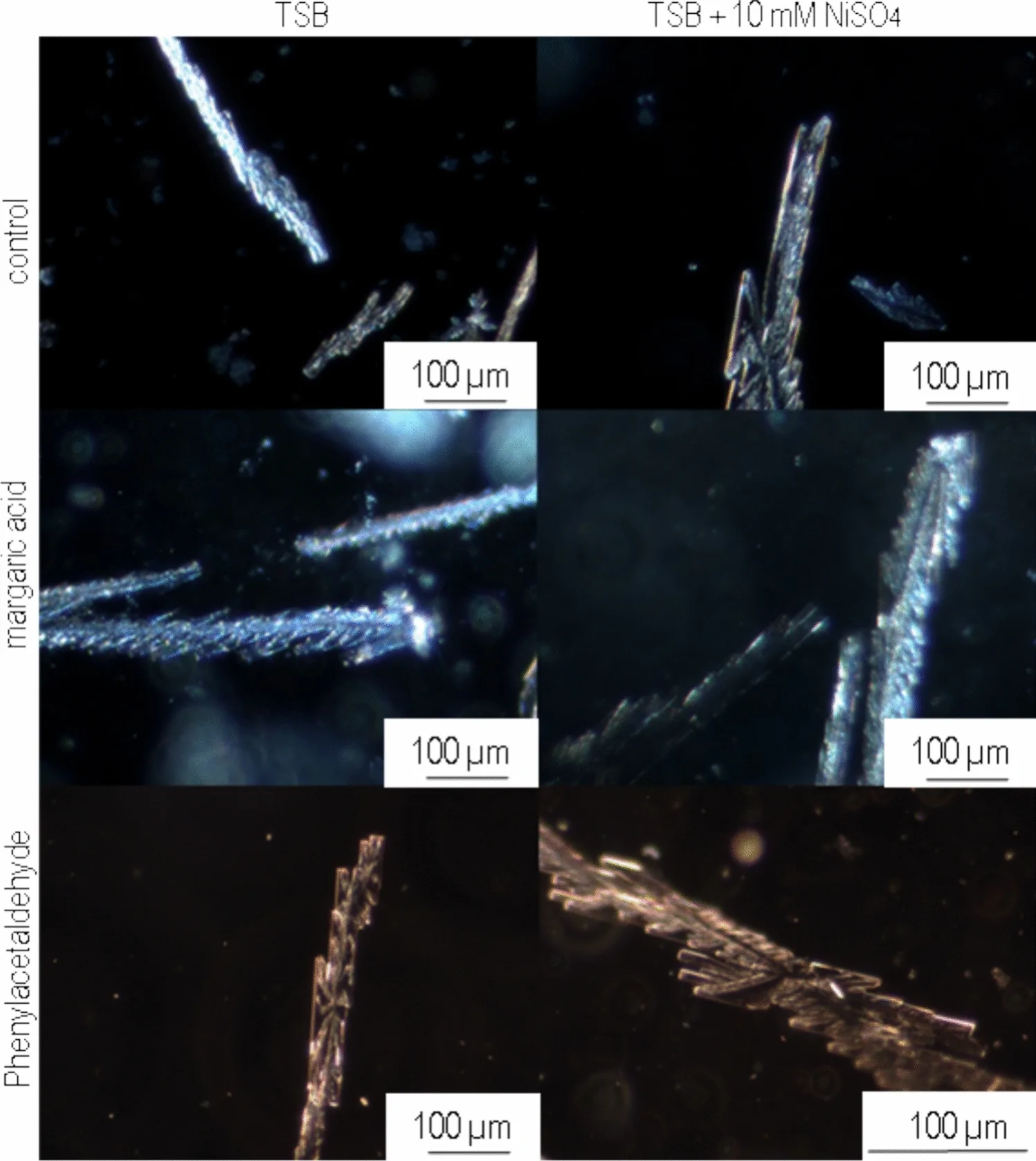
Morphology of minerals. Biominerals were precipitated in the presence of phenylacetaldehyde or margaric acid and compared to control supernatant without the added substances. No relevant difference in morphology could be detected

A more detailed approach was required to detect single secreted molecules with different abundances in the same sample before and after precipitation. Such a compound might have direct influence on determining the (macro)morphology of the minerals. In the context of biomineralization, few molecules were observed to be removed from the supernatant during precipitation of the minerals produced by *S. mirabilis* P16B-1. Ergothioneine, N,N-diacetyllegionaminic acid, nevaltophin F, and 1,5-diaminonaphthalene (1,5-DAN) were found to co-precipitate from the supernatant of *S. mirabilis* P16B-1 only when nickel-containing minerals had been formed. On the other hand, phenylacetaldehyde, margaric acid, and 2-(furan-2—yl)−6-(2S,3S,4-trihydroxybutyl)pyrazine were reduced from the supernatant when struvite or Ni-struvite was formed (Online Resource 3). To exclude a role of the latter compounds, commercially available phenylacetaldehyde and margaric acid were applied to a mock solution with subsequent mineralization. However, no substantial differences were observed in the morphology of crystals (Fig. 3), leaving ergothioneine, diacetyllegionaminic acid, nevaltophin F, and diaminonaphthalene as potential candidates influencing macromorphology of Ni-struvite.

### The Contribution of Proteins to Mineralization

**Fig. 4:**
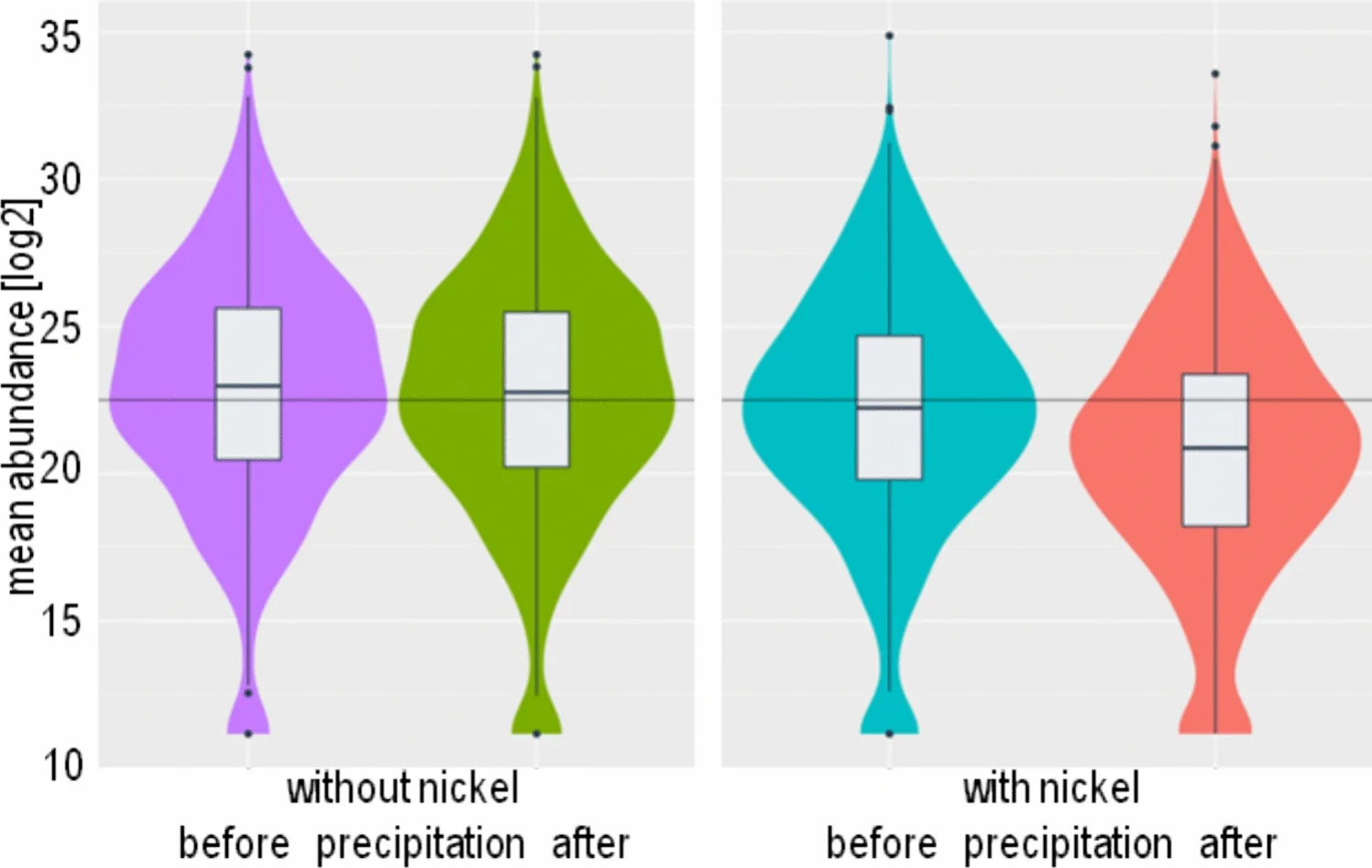
Proteome changes upon mineral formation. The proteome changes (log2-fold) are shown with a Violin Plot to reveal significant changes depending on treatment. The main abundances of the proteins in culture supernatant showed significant differences between the samples before and after mineral formation occurred only when media contained additional nickel (10 mM NiSO_4_, right panel)

Since proteins also are specifically excreted, their role was similarly addressed. The proteins in the two treatments significantly differed from each other, reflecting specific cellular responses to nickel. The contribution of proteins present in the sterile filtered supernatant to struvite and Ni-struvite precipitation revealed that without nickel, the secretomes showed no clear separation of samples before or after the precipitation of minerals, and the majority (99.4%) did not change in concentration after mineral precipitation significantly. This changed for the secretome in nickel treatments that showed a clear separation before and after mineral precipitation, and here, 74% proteins decreased in concentration following Ni-struvite precipitation (Fig. 4; the full dataset is available at https://www.ebi.ac.uk/pride; dataset identifier PXD049698).

Under conditions of Mg-struvite precipitation, i.e., without excess Ni in the growth medium, 5 proteins of the 1216 identified proteins were reduced in abundance after struvite precipitation. However, *p-*values for all of these were above 0.01. Thus, the changes of these few proteins were significant, but not highly significant.

Again, looking for loss of protein from solution specifically in nickel-containing Ni-struvite mineralizing samples, 1054 proteins were identified in the culture supernatant, with 740 decreasing in abundance after mineral precipitation. Of those, 18 proteins with highly significant abundance change and high loss were further analyzed for secretion signals (Table 1). This selection of actively excreted proteins considered potentially involved in Ni-struvite morphogenesis yielded 10 proteins, all of which were only changing significantly when nickel-containing struvite was precipitated, while abundances did not change significantly in case of Mg-struvite precipitation.

**Table 1:** Proteins with high abundance and high co-precipitation during Ni-struvite mineralization. Total and % loss as well as statistical significance are given and the prediction for secretion pathway is indicated

Acc #	Protein	Total loss	% loss	p(a vs b)
		+Ni-Ni	+Ni-Ni	+Ni-Ni
QUW81028.1	CU044_5270 family protein	9,26E+081,89E+07	72,629,7	0,0010,01
QUW77912.1	Xaa-Pro dipeptidyl-peptidase	1,73E+091,35E+06	69,10,1	0,0020,98
QUW84500.1	tyrosinase family protein	3,60E+09-3,89E+08	67,4-2,7	0,0030,84
QUW81201.1	TerD family protein	1,00E+092,33E+08	66,33,1	0,0010,96
QUW78058.1	TerB family tellurite resistance protein	8,06E+084,81E+08	64,010,5	0,0010,78
QUW77913.1	M1 family metallopeptidase	8,30E+08-1,78E+07	63,1–2,2	0,0010,84
QUW83741.1	TerD family protein	6,62E+082,86E+08	61,94,5	0,0010,92
QUW84845.1	S8 family peptidase	8,68E+082,31E+07	61,71,7	0,0020,97
QUW80326.1	cold-shock protein	9,05E+081,77E+08	61,113,1	0,0010,80
QUW79647.1	ABC transporter substrate-binding protein	6,17E+088,93E+07	60,45,9	0,0050,80
QUW82652.1	5'-nucleotidase containing protein	6,27E+08-7,24E+06	60,4-0,7	0,0010,95
QUW82667.1	lytic murein transglycosylase	7,79E+08-1,59E+07	59,7-3,1	0,0010,84
QUW80952.1	SCO family protein	3,43E+091,76E+08	59,46,6	0,0010,76
*QUW79797.1	NikA, ABC transporter substrate-binding	1,85E+10-1,62E+07	59,2-0,1	0,0011,0
QUW80793.1	D-alanyl-D-alanine carboxypeptidase	1,49E+091,62E+07	59,11,3	0,0010,98
QUW79064.1	HPr family phosphocarrier protein	8,86E+089,29E+08	57,213,1	0,0020,68
QUW84173.1	penicillin acylase family protein	9,33E+08-1,45E+08	54,6–18,6	0,0030,73
QUW78179.1	sugar phosphate isomerase/epimerase	1,12E+09-4,68E+08	52,9–21,6	0,010,71

*Sec/SPI* standard secretory signal peptides with Sec translocon transport and signal peptidase I cleavage; *Sec/SPII* lipoprotein signal peptides transported by Sec and cleaved by signal peptidase II; *Tat/SPI* TAT translocon transport cleaved by signal peptidase I; * highlights NikA

Among the 10 genes potentially involved in Ni-struvite formation, three (metallo)proteases/peptidases (QUW77912, QUW77913, QUW80793) and two ABC transporter-associated substrate binding proteins (QUW79647, QUW79797) were present. This seems to indicate a specific enrichment for metal binding proteins. The proteins with highest statistical significance of being coprecipitating during mineral formation were classified with respect to cellular processes using the KEGG classification tool (Online Resource 4). One gene annotated to encode a nickel binding protein, NikA (QUW79797), was further analyzed bioinformatically.

### Indications for Involvement of NikA

**Fig. 5:**
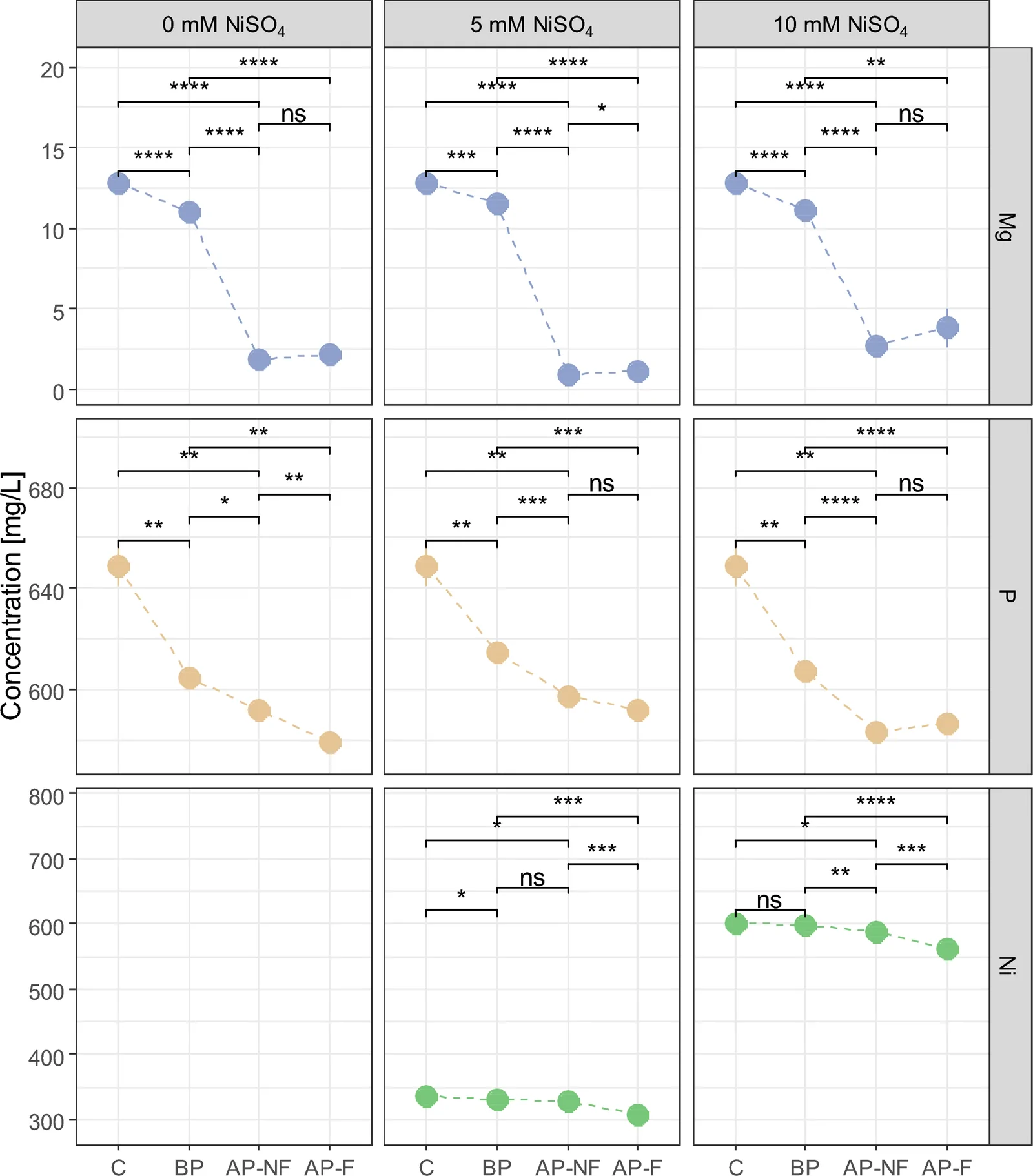
Biomineral formation in dependence of mineral and biomolecule availability. Concentration of Mg, P, and Ni in the supernatant of *S. mirabilis* P16B-1 before mineral precipitation (BP) and after precipitation (AP) were compared to medium before cultivation (C). Samples were also subjected to ultrafiltration using 30 kDa MWCO filters (AP-F) or not filtered (AP-NF). While during growth, relevant amounts of phosporus were taken up from the medium, growth did not significantly reduce the Mg contents. These, as well as Ni when available, were only removed from the liquid phase after mineralization had taken place. Statistical significance: ns, non-significant; *, *p* < 0.05; **, *p* < 0.01; ***, *p* < 0.0001

Since proteins were not essential for crystal formation as seen with size exclusion above, a role in determining nickel incorporation was assessed. Therefore, the concentrations of Mg and Ni in the supernatant were determined after mineral precipitation. By removing proteins larger than 30 kDa, an indirect means of assessing their involvement in biomineralization could be obtained. The concentration of Mg after mineral precipitation exhibited no significant difference between samples subjected to ultrafiltration and the unfiltered ones, suggesting that the amount of struvite precipitated was consistent in both samples (Fig. 5). In contrast, the concentration of nickel after mineral precipitation in filtered samples decreased with high significance compared to the non-filtered ones. Thus, a role for proteins in nickel incorporation could be corroborated.

As seen with the secretome analysis above, a protein potentially involved in nickel supply is the nickel binding NikA, which has around 58 kDa in *S. mirabilis* P16B-1. In Gram-negative bacteria, this nickel binding domain supplies the metal to the ABC transporter with the subunits NikBCDE inserted into the inner membrane. The *S. mirabilis* P16B-1 genome contains several copies encoding NikA. The protein sequence identified here was found in an operon with duplicated genes *nikABCDE* (Online Resource 5). Instead of the regulatory protein NikR located downstream of the gene cluster in *E. coli, S. mirabilis* P16B-1 features an oxygen chelator family protein in the genomic region. The protein contains 541 amino acids with a molecular mass of 58.3 kDa and a net negative charge (GRAVY index after Dyte and Doolittle of −0.403). While overall the protein is rather hydrophilic, the N-terminus is intrinsically disordered (compare Online Resource 6) and contains a signal sequence for excretion. It is predicted to be stable with a half-life of more than 10—30 h. Its pI is alkaline at pK = 8.71 because of the high number of charged amino acids (63 negatively and 57 positively charged amino acids). The alphafold prediction reveals an intrinsically disordered N-terminus of 34 amino acids that may facilitate binding to mineral surfaces.

## Discussion

The formation of struvite and Ni-struvite by *S. mirabilis* P16B-1 is differentially influenced, with Ni-struvite showing bacterial impact on macro-morphologies [37]. The differences observed in minerals formed abiotically to the ones produced by *S. mirabilis* P16B-1 were linked to candidate organic molecules and proteins produced and secreted by this bacterium. To achieve this step forward in the newly described bacterially influenced biomineralization, the metabolome and proteome of culture supernatants were evaluated before and after mineral precipitation.

In the metabolome analysis, the low separation of samples did not suggest a major role. However, four compounds (namely ergothioneine, diacetyllegionaminic acid, nevaltophin F, and diaminonaphthalene) cannot be ruled out for a contribution to mineral growth pattern. A potential role might be envisioned by releasing some of the lattice stress that results in nickel struvite, for nickel having a smaller ion radius as compared to magnesium.

The general changes in the excreted metabolites of nickel versus no nickel grown cell was to be expected, since metal stress in bacteria leads to a metabolic reprogramming in energy, amino acid, and lipid metabolism [48]. Some of the observed compounds might directly be involved in heavy metal resistance by acting as chelators, which has been described for melanin [49, 50] or siderophores [51–53]. Metal binding molecules can either immobilize Ni ions, preventing their incorporation into the mineral structure, or they can act as carriers, facilitating the delivery of the metal to the growing mineral and thereby aiding in mineral formation [32, 54, 55]. Ergothioneine is a mercapto-derivative of histidine synthesized by several actinobacteria with known anti-oxidative traits [54–56]. Thus, its excretion likely triggered by nickel-dependent oxidative stress, and it may be involved in protecting the ammonia that is needed for incorporation into the growing crystal lattice under oxic conditions. Diaminolegionamic acid is found in the outer membrane of Gram-negatives. However, a synthetase has been identified within a dudomycin biosynthesis cluster in *Streptomyces* [57].

Like legionellic acid, the indole compound nevaltophin F features several keto groups. For both, a potential role in nickel binding (and delivery) thus may be possible. Polymers of the naphthalene compound 1,5-DAN have shown strong absorption capability of metals, including Pb^2+^, Hg^2+^, and Ag^+^ [58]. In our investigation, however, the potential to preferentially bind to growing crystalloids would be prerequisite for changed morphology.

Like with metabolites, proteins might specifically interact with one or two faces of the growing crystalloid. This might involve more passive adsorption of the protein to one face preferentially, or even more specific binding.

The lack of evidence for proteins impacting Mg-struvite is in accordance with our finding that only Ni-struvite macromorphology was variable. For Mg-struvite with *Proteus mirabilis,* coffin-shaped struvite biominerals have been observed [59], while other biomimetic analyses showed mainly needles or rods [60]. Ni-struvite from wastewaters has shown needle, rod, plate, or prism shapes [11]. This aligns nicely with the concept of non-conventional biomineralization, and very much resembles structures observed in our case. Hence, the impact of secreted proteins was investigated. Here, secreted proteins were highly contributing to differentiating the samples. This is in accordance with differential binding to (faces of) the growing minerals.

In media containing nickel, *S. mirabilis* P16B-1 produced more minerals than the control without the metal [36]. At the same time, nickel in the medium leads to increased expression of NikA. While a clear stoichiometry cannot be obtained at this point, the clear correlation indicates a role that is relevant to crystal growth rather than nucleation (compare [36]). Since proteins interact with the surface of minerals, a delivery of nickel sequestered to the protein onto the mineral surface seems possible.

Three pathways for secretion were predicted for the 10 selected proteins. In addition to the sec translocon, twin arginine transport (TAT pathway) of folded, co-factor containing proteins was predicted. Two signal peptidases were associated with the sec transport pathway, while only signal peptidase I was predicted to release the TAT-translocated proteins.

The ten secreted proteins with high loss during mineral formation showed enrichment for proteins containing metal binding sites, with three (metallo) proteases as well as two transport-associated substrate binding proteins. In addition, a 5'-nucleotidase domain protein, a penicillin acylase family protein, and a sugar phosphate isomerase/epimerase as well as one more cell wall-associated protein, a lytic murein transglycosylase, as well as a protein involved in cytochrom C synthesis might also have specific preferences for one face of the growing Ni-struvite. The identified peptidases belong to the Xaa-Pro dipeptidyl-peptidases and M1 family metallopeptidases and specify one cell wall-specific D-Ala/D-Ala carboxypeptidase. The two ABC transporter substrate binding proteins show good conservation, with one being related to a known nickel binding protein. This shows a high prevalence of metabolism and enzyme classes.

To comply with the current non-canonical biomineralization model, an intrinsically disordered structure would be predicted for these proteins [15, 16, 61]. The high prevalence of peptidases indicates that processing of one or more of the excreted proteins might contribute to releasing the needed, unstructured polypeptides.

NikA as a nickel binding protein has been described associated with an ABC transporter. In *E. coli*, this operon contains, in addition to the proteins that constitute the transporter, a regulatory protein that represses the expression of the operon in the presence of high concentrations of nickel [62]. This function mediates protection against excess influx of the metal at toxic levels. *S. mirabilis*, instead, features a VOC family protein at a similar genomic position. This protein family of metalloenzymes can perform a wide range of reactions, but a regulatory function was not proposed [63].

In *S. coelicolor*, the regulation of *nikA* is dependent on the nickel-uptake regulator *nur* (see [47]). For *S. mirabilis* P16B-1, a transcriptional repressor with high similarity could be identified (Online Resource 7). This seems important in the context of NikA being up-regulated in the presence of nickel. Hence, the general transcriptional control facilitating expression through the *nur*-dependent DNA binding will activate transcription of NikA in a nickel-dependent way that is triggered already at femtomolar intracellular nickel concentrations [47, 64]. Only in the presence of nickel, amounts of NikA necessary for co-precipitation are excreted. This can be easily facilitated through the pre-existing regulatory *nur* network involved in intracellular nickel homeostasis. Additionally, the sequence of NikA from *S. mirabilis* P16B-1 contains a motif for a lipoprotein peptide signal sequence indicating secretion (probability 0.994; Online Resource 8).

## Conclusion: Microbially Influenced Biomineralization as a Concept

**Fig. 6:**
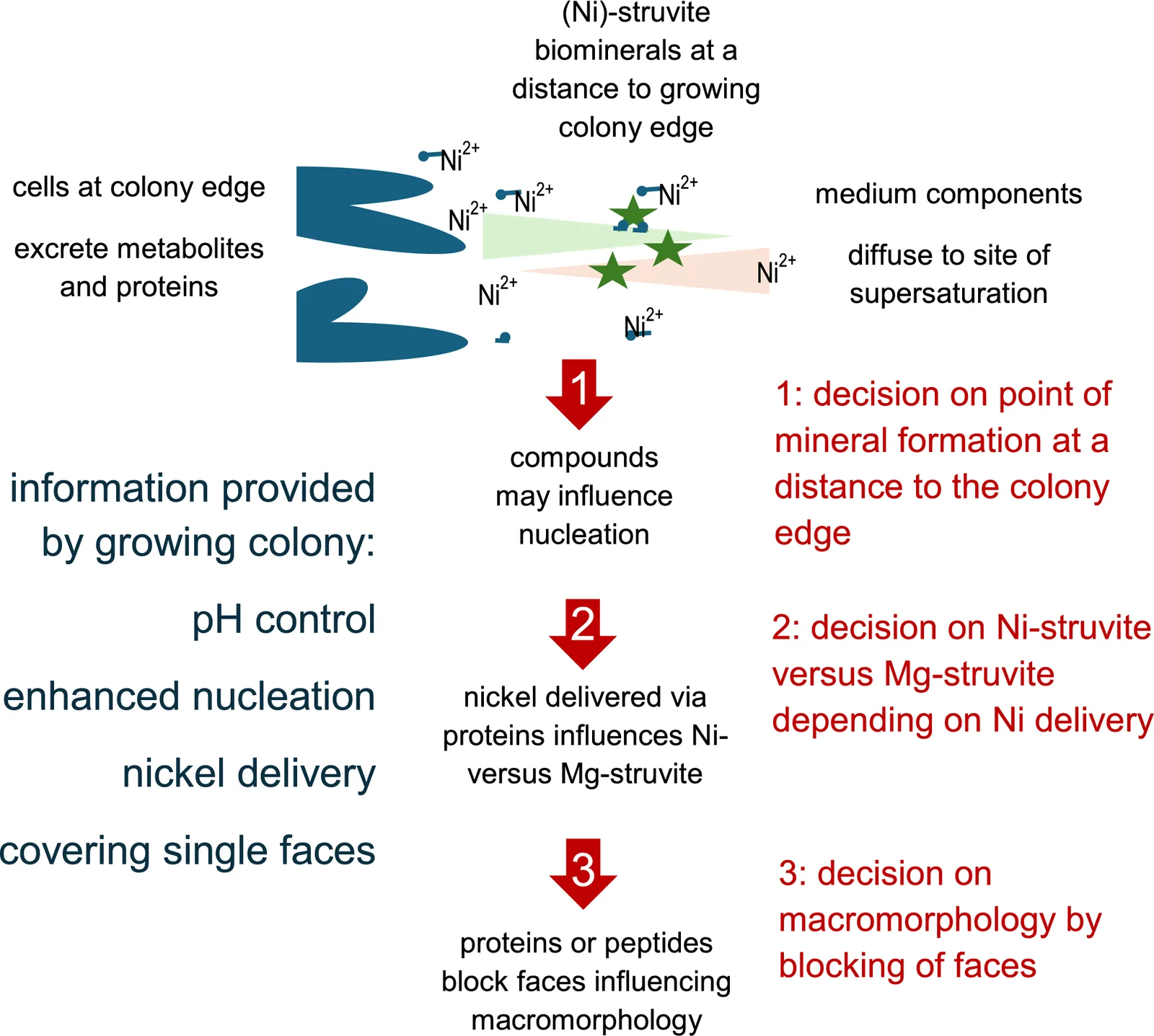
Concept of biomineral formation involving active control in the microbially induced struvite or Ni-struvite precipitation. While organic molecules are involved in nucleation, the metastable aggregates are shaped by incorporation of proteins. In addition, delivery of Ni ions is promoted, facilitating the decision between Ni-struvite or conventional Mg-struvite biomineralization

The decrease in concentration of proteins in the remaining liquid suggested that they may bind to the mineral during mineral growth which might allow for different macro-morphologies of the resulting minerals. This inspired the hypothesis of microbially influenced biomineralization that goes beyond providing crystallization point and allowing for crystal growth in a preferred direction (Fig. 6). Three steps are under control of the microbe, the first active process being the excretion of metabolites that may be involved in enhanced nucleation. Then, the binding of nickel to NikA, usually necessary to feed the nickel transporter, can diffuse and permit binding to a crystalloid aided by the intrinsically disordered N-terminal tail. Upon adsorption to the mineral, a structure may be stabilized that releases nickel which therefore becomes locally concentrated in a gradient of enriched nickel surrounding the colony, while the essential phosphate is depleted surrounding growing cells. At the point where Ni-struvite is supersaturated, crystalloids may grow. The enzymes like excreted urease in addition aid crystallization with a pH gradient surrounding the colony. The constant delivery of nickel to the growing crystalloids via NikA will influence the decision of Mg versus Ni incorporation. The preferential binding of NikA or a proteolytically formed peptide to onto one face of the mineral surface will influence the direction into which the crystal enlarges. Altogether, with these steps the cells exert an active rather than passive influence on the site of mineral precipitation not directly at the cell surface, the mineralogy, as well as mineral micromorphology. This concept is combining earlier concepts that differentiated between active and more passive roles like microbially controlled and microbially induced mineralization as well as the roles of microbes in non-canonical mineralization.

The nickel binding protein NikA, the biomineralization of Ni-struvite with *S. mirabilis* P16 presents a good model to further study the processes underlying specific biomineral formation in a system that adds to current concepts provided from calcite mineral formation. With this concept, we hope to add to the growing evidence of a more active role of microbes in biomineralization by contributing to a more molecular understanding of microbially influenced biomineralization. While NikA involvement should be restricted to Ni-struvite formation, Co or Zn containing struvites might share some of the features proposed here. The investigation involving formation of Mg or Ni containing crystals with a highly metal-resistant actinobacterium allowed us to extend the current concept that has been proposed from investigations of bone, calcite or magnetite biomineralization, which have been the most prominent examples of study so far.

The findings open roads to potential biotechnological applications. First, the biomineralization of nickel struvite constitutes a resistance factor for heavy metal contaminated soils. This has been stressed before and inspired the use of metal-resistant microbes in bioremediation approaches for metal-rich soils [37, 65–67]. However, in case of struvite versus nickel struvite precipitation, an application during wastewater treatment can be easily imagined [10, 34, 68].

## Data Availability

Not applicable to the present study.
